# EGFR Inhibition in a Pretreated Sacral Chordoma: A Role for Erlotinib? Case Report and a Brief Review of Literature

**Published:** 2017-07-01

**Authors:** D. Trapani, F. Conforti, T. De Pas

**Affiliations:** 1 University of Milan, Department of Oncology and Hematology, Via Festa del Perdono 7, Milan, Italy; 2 Early Drug Development for innovative therapies, European Institute of Oncology, Via Ripamonti 435, Milan, Italy; 3 Melanoma, sarcoma and rare tumors program, European Institute of Oncology, Via Ripamonti 435, Milan, Italy

**Keywords:** chordoma, erlotinib, Imatinib refractory, sacral tumor

## Abstract

We describe the case of a 69-year old male with an EGFR- positive Imatinib refractory sacral chordoma with synchronous lung metastases, treated with erlotinib, a first-generation EGFR inhibitor. After disease progression following first-line Imatinib and a combination therapy with everolimus plus metformin, we made a challenge with an EGFR tyrosine kinase inhibitor (EGFR TKI), erlotinib. Despite a brief clinical benefit, the patient presented a rapid clinical deterioration leading to death, after 8 weeks of treatment.

## CASE REPORT

A 69-year old man was referred to our institution for a refractory sacral chordoma.

First diagnosis was performed in April 2012 through an incisional biopsy of the primitive tumor; at presentation, synchronous lung metastases were radiologically detected. The primitive mass was surgically resected *en-bloc* with distal sacrum (distal sacrectomy): pathology report confirmed the presence of a 12, 5 cm chordoma, infiltrating the surrounding soft tissues and the overlying skin with ulceration. IHC (immunohistochemistry) revealed a Brachyury, PDGFR-β and EGFR 2+ positive tumor (R pharmDx™ Dako kit) with evidence of pS6 phosphorylation in more than 70% of cells.

He received a first-line systemic therapy with a tyrosine kinase inhibitor (TKI), Imatinib 400mg, in another institution; everolimus was added to imatinib for a slight progression of disease (PD) after 4 months, reaching a disease stability (SD) for 16 months; the TKI was eventually substituted with metformin with SD.

The patient was then referred to our institution: he presented a rapid clinical deterioration of the clinical status with dyspnea on exertion; a low flow oxygen-therapy on demand was so provided. We performed a ^18^FDG-PET evaluation on July 2015 that revealed lung, bone and pelvic diffuse pathological accumulations. Afterwards, in the absence of therapeutic alternative options for a good performing patient, a challenge with an EGFR inhibitor, Erlotinib 150mg daily, was proposed.

A better tolerance to exercise with a reduction of dyspnea on exertion and no more continuous, daily demand of oxygen support was reported. However, despite a transient clinical benefit, the patient presented a new rapid clinical deterioration after 8 weeks of treatment, leading to death in September 2015.

## DISCUSSION

Chordoma is a rare mesenchymal tumor arising from the remnants of notochord, an embryonic midline structure common to all *Chordata*- *Phylum* members. In higher vertebrates including humans, the notochord gets ossified and contributes to the formation of vertebrae, persisting as nucleus pulposus of intervertebral discs (1); however, microscopic foci can remain in the cranial and caudal portion of the spine. Chordoma is a rare bone cancer, accounting 1–4% of all bone malignancies; it arises mainly from the axial skeleton, affecting most commonly cranial and caudal bone midline sites like sacrum and skull base (2).

The clinical behavior of this tumor is insidious for the unpredictable possibility of spread on bone and neural structures; moreover, the indolent and clinically silent course delays the diagnosis until advanced disease. Mostly, spine chordomas involve S4–S5 or coccygeal region protruding anteriorly into the pelvis with late onset neurological symptoms, due to neural roots compression (3). Chordoma rarely presents as metastatic at first diagnosis: OS seems to be poorly affected by metastatic progression and local control appears to be correlated more to the prognosis (4).

The backbone of therapy for the localized disease is surgery with wide margins without violation of tumor capsule, with a significant impact on DFS and OS (5).

Concerning medical treatment, chordoma seems to be insensitive to cytotoxic chemotherapy; in a series of 33 cases published by Azzarelli (6), no chemotherapeutic regimen induced a significant tumor respons though a potential response to continuous infusion ifosfamide was demonstrated in high-grade de-differentiated tumors (7) as well as with combination regimen containing anthracyclines, platinum-compounds and alkylating drugs (8).

This intrinsic chemo-resistance paves the way to different anti-tumor approaches, designed from biologic features and molecular characterization of chordoma. Kilgore S. showed a high expression of cyclin D1, a key regulator of cell cycle-progression in a series of 26 chordoma samples (9); overexpression of CDK4, p53 and MDM2 was detected in 20–56% of sacral and skull-base chordoma, with a poor prognostic significance (10). Membrane growth factor receptors (TKRs) were demonstrated to be highly expressed in chordomas: scatter factor/hepatocyte growth factor receptor (c-Met), epidermal growth factor receptor (EGFR/HER1) and different phosphorylated platelet-derived growth factor receptors (PDGFRs) seem to play a key role in tumor proliferation (11); this suggests a potential role of tyrosine-kinase receptor inhibitor as therapeutics. Hence, a high-level copy number gain of 7p12 band (EGFR locus) and EGFR expression (67–81%) have been demonstrated; TKRs (PDGFR-a, c-Met, EGFR) are variously expressed in 97% of the recurrence samples analyzed (12).

Furthermore, this molecular *stigmata* are potentially *druggable*. TKIs targeting angiogenesis have shown to be active against chordoma; this may probably reflect a cross-inhibition of different overlapping molecular patterns (13). On this subject, Casali et. al detected an anti-tumor effect in term of tumor liquefaction, metabolic and clinical response of imatinib mesylate 800mg daily; he supposed an activity mediated by PDGFRβ-inhibition (14). Similarly, a response to sunitinib 37,5 mg daily was demonstrated in a multicenter phase-II trial: 44% of chordoma patients achieved a SD concurrent to a qualitative decrease in tumor density; this pattern of response is considered to be characteristic of tumor response to VEGF-directed therapy (15). Additionally, EGFR-inhibitors have shown a clinical activity, particularly in the second-line setting after imatinib failure (16). Results from a phase II trial reported a modest antitumor activity of lapatinib administered at 1500mg continuously until progression or unacceptable toxicity in advanced EGFR-positive chordomas; the clinical benefit rate was 22,2% by RECIST for the intention-to-treat population (17).

Siu evaluated the activity of erlotinib on a patient-derived chordoma xenograft; he showed a strong *druggable* activation of EGFR: exposure of tumor cells to erlotinib resulted in a dimensional anti-tumor response concurrent to a reduction of phosphorylation of Tyr845 residue, thus supporting a favorable clinical usage (18).

Based on this molecular notion, a compassionate use of Erlotinib for relapsed-refractory chordoma can be proposed. Few recent case reports showed a clinical activity of erlotinib (Tab 1) but its benefit has not been evaluated in a prospective trial. Indeed, a sustained response to erlotinib of a clivus-arising chordoma after imatinib failure has been described by Houessinon A. (19): a 28 months continuous partial-response was reported, the longest described in literature in this setting.

## CONCLUSION

Our case report shows no clear clinical response to erlotinib in an imatinib-refractory sacral chordoma. Few clinical data are available to support the choice of Erlotinib in this setting, with discordant experiences reported. Therefore, a prospective trial comparing erlotinib Vs. standard therapy in different setting is warranted, considering the high rate of EGFR- positive chordomas and preclinical encouraging data. Furthermore, EGFR mutational status prognostic significance and predictive role should be clarified.

## Figures and Tables

**Table 1:** Cases of chordoma treated with erlotinib 150mg single-agent.

Sex	Localization	Previous treatment	EGFR status (other molecular features)	EGFR status assessment	Type of Response	Duration of response *(months)*	Reference
Male	Sacrum	Sur: distal sacral and coccygeal resection RT: 66Gy for local relapse Sur and imatinib 600mg CYT997 (Lexibulin)	EGFR WT (PDGFR-β WT)	EGFR 18–24 exon sequencing FISH for EGFR and PDGFR-β	PR	11 OG	Singhal N, 2009 [20]
Male	Sacrum	Sur: distal sacral and coccygeal resection Post-op 60Gy RT Bone metastasis resection and RT Imatinib 400mg Imatinib 600mg	EGFR WT (chromosome 7 trisomy in 38% of the tumor cells)	IHC: 90% EGFR positive tumor cells EGFR, KRAS, BRAF, KIT, PDGFR-α sequencing FISH for EGFR	PR	12	Launay SG, 2011 [16]
Woman	Clivus	Sur: transphenoidal tumor resection Proton therapy 68Gy Imatinib 400mg Imatinib 800mg	Not reported	Not reported	PR	28 OG	Houessinon A, 2015 [19]

Sur: surgery; RT: radiotherapy; PR: partial response according to RECIST criteria; WT: wild type; OG: response ongoing at time of case report writing; post-op: post-operative; IHC: immunohistochemistry; FISH: fluorescence in-situ hybridization; EGFR: epidermal growth factor receptor; PDGF: platelet-derived growth factor.

## References

[1] Stemple DL (2005). Structure and function of the notochord: An essential organ for chordate development. Development.

[2] Chugh R (2007). Chordoma: The Nonsarcoma Primary Bone Tumor. The Oncologist.

[3] Bjornsson J, Wold LE, Ebersold MJ, Laws ER (1993). Chordoma of the mobile spine. A clinicopathologic analysis of 40 patients. Cancer.

[4] Boriani S, Chevalley F, Weinstein JN (1996). Chordoma of the spine above the sacrum. Treatment and outcome in 21 cases. Spine.

[5] Jawad MU, Scully SP (2010). Surgery significantly improves survival in patients with chordoma. Spine (Phila Pa 1976).

[6] Azzarelli A, Quagliuolo V, Cerasoli S (1988). Chordoma: natural history and treatment results in 33 cases. J Surg Oncol.

[7] Fleming GF (1993). Dedifferentiated chordoma. Response to aggressive chemotherapy in two cases. Cancer.

[8] Hanna SA (2008). Dedifferentiated chordoma: a report of four cases arising ‘de novo’. J Bone Joint Surg Br.

[9] Kilgore S, Prayson RA (2002). Apoptotic and proliferative markers in chordomas: a study of 26 tumors. Ann Diagn Pathol.

[10] Yakkioui Y, Temel Y, Creytens D, Jahanshahi A, Fleischeuer R, Santegoeds R, Van Overbeeke JJ (2013). A Comparison of Cell-Cycle Markers in Skull Base and Sacral Chordomas. World neurosurgery.

[11] Weinberger PM, Yu Z, Kowalski D, Joe J, Manger P, Psyrri A, Sasaki CT (2005). Differential expression of epidermal growth factor receptor, c-Met, and HER2/neu in chordoma compared with 17 other malignancies. Arch Otolaryngol Head Neck Surg.

[12] Gulluoglu S, Turksoy O, Kuskucu A, Ture U, Bayrak OF (2015). The molecular aspects of chordoma. Neurosurg Rev.

[13] Walcott BP, Nahed BV, Mohyeldin A, Coumans JV, Kahle KT, Ferreira MJ (2012). Chordoma: current concepts, management, and future directions (Review article). Lancet Oncol.

[14] Casali PG, Messina A, Stacchiotti S, Tamborini E, Crippa F, Gronchi A, Orlandi R, Ripamonti C, Spreafico C, Bertieri R, Bertulli R, Colecchia M, Fumagalli E, Greco A, Grosso F, Olmi P, Pierotti MA, Pilotti S (2004). Imatinib mesylate in chordoma. Cancer.

[15] George S, Merriam P, Maki RG, Van den Abbeele AD, Yap JT, Akhurst T, Harmon DC, Bhuchar G, O’Mara MM, D’Adamo DR, Morgan J, Schwartz GK, Wagner AJ, Butrynski JE, Demetri GD, Keohan ML (2009). Multicenter Phase II Trial of Sunitinib in the Treatment of Nongastrointestinal Stromal Tumor Sarcomas. J Clin Oncol.

[16] Launay SG, Chetaille B, Medina F, Perrot D, Nazarian S, Guiramand J, Moureau-Zabotto L, Bertucci F (2011). Efficacy of epidermal growth factor receptor targeting in advanced chordoma: case report and literature review. BMC Cancer.

[17] Stacchiotti S, Tamborini E, Lo Vullo S, Bozzi F, Messina A, Morosi C, Casale A, Crippa F, Conca E, Negri T, Palassini E, Marrari A, Palmerini E, Mariani L, Gronchi A, Pilotti S, Casali PG (2013). Phase II study on lapatinib in advanced EGFR positive chordoma. Ann Oncol.

[18] Siu IM, Ruzevick J, Zhao Q, Connis N, Jiao Y, Bettegowda C, Xia X, Burger PC, Hann CL, Gallia GL (2013). Erlotinib inhibits growth of a patientderived chordoma xenograft. PLoS One.

[19] Houessinon A, Boone M, Constans JM, Toussaint P, Chauffert B (2015). Sustained response of a clivus chordoma to erlotinib after imatinib failure. Case Rep Oncol.

[20] Singhal N, Kotasek D, Parnis FX (2009). Response to erlotinib in a patient with treatment refractory chordoma. Anticancer Drugs.

